# Lung Cancer Incidence and Long-Term Exposure to Air Pollution from Traffic

**DOI:** 10.1289/ehp.1002353

**Published:** 2011-01-12

**Authors:** Ole Raaschou-Nielsen, Zorana Jovanovic Andersen, Martin Hvidberg, Steen Solvang Jensen, Matthias Ketzel, Mette Sørensen, Steffen Loft, Kim Overvad, Anne Tjønneland

**Affiliations:** 1 Institute of Cancer Epidemiology, Danish Cancer Society, Copenhagen, Denmark; 2 Department for Atmospheric Environment, National Environmental Research Institute, Aarhus University, Roskilde, Denmark; 3 Section of Environmental Health, Department of Public Health, University of Copenhagen, Copenhagen, Denmark; 4 Department of Epidemiology, School of Public Health, Aarhus University, Aarhus, Denmark

**Keywords:** air pollution, lung cancer, traffic

## Abstract

**Background:**

Previous studies have shown associations between air pollution and risk for lung cancer.

**Objective:**

We investigated whether traffic and the concentration of nitrogen oxides (NO_x_) at the residence are associated with risk for lung cancer.

**Methods:**

We identified 592 lung cancer cases in the Danish Cancer Registry among 52,970 members of the Diet, Cancer and Health cohort and traced residential addresses from 1 January 1971 in the Central Population Registry. We calculated the NO_x_ concentration at each address by dispersion models and calculated the time-weighted average concentration for all addresses for each person. We used Cox models to estimate incidence rate ratios (IRRs) after adjustment for smoking (status, duration, and intensity), environmental tobacco smoke, length of school attendance, occupation, and dietary intake of fruit.

**Results:**

For the highest compared with the lowest quartile of NO_x_ concentration at the residence, we found an IRR for lung cancer of 1.30 [95% confidence interval (CI), 1.05–1.61], and the IRR for lung cancer in association with living within 50 m of a major road (> 10,000 vehicles/day) was 1.21 (95% CI, 0.95–1.55). The results showed tendencies of stronger associations among nonsmokers, among those with a relatively low fruit intake, and among those with a longer school attendance; only length of school attendance modified the effect significantly.

**Conclusions:**

This study supports that risk for lung cancer is associated with different markers of air pollution from traffic near the residence.

Lung cancer is one of the most frequent cancers and has a poor prognosis. Active tobacco smoking is the major cause, but certain occupational exposures, residential radon, environmental tobacco smoke (ETS), and lower socioeconomic status are also established risk factors (Spitz et al. 2006).

Several cohort and case–control studies have indicated higher risk for lung cancer in association with different measures of exposure to ambient air pollution. Ambient concentrations of sulfur dioxide and particulate matter (PM) with an aerodynamic diameter ≤ 10 μm (PM_10_) was associated with lung cancer incidence in a cohort of nonsmoking California adults (Beeson et al. 1998), whereas fine PM with an aerodynamic diameter ≤ 2.5 μm (PM_2.5_) was associated with lung cancer mortality in two other cohort studies from the United States (Laden et al. 2006; Pope et al. 2002). A joint European study of nonsmokers showed associations between lung cancer incidence and nitrogen dioxide (NO_2_) (Vineis et al. 2006); three Scandinavian studies showed associations with model-estimated nitrogen oxides (NO_x_) and NO_2_ (Nafstad et al. 2003; Nyberg et al. 2000; Raaschou-Nielsen et al. 2010); and a Dutch study showed associations with black smoke and residence near traffic (Beelen et al. 2008). Furthermore, diesel engine exhaust causes cancer in experimental animals [International Agency for Research on Cancer (IARC) 1989], and studies show a higher risk for lung cancer among populations occupationally exposed to diesel engine exhaust (Bhatia et al. 1998; Garshick et al. 2008; Lipsett and Campleman 1999). Thus, the overall picture is an increased risk for lung cancer in association with various measures of exposure to air pollution, with a strength of association for traffic-related air pollution comparable with that of ETS (Vineis et al. 2007).

Further, several studies have indicated that the effects of air pollution on the risk for lung cancer might be modified by smoking status (Beelen et al. 2008; Nyberg et al. 2000; Pope et al. 2002; Yorifuji et al. 2010), fruit consumption (Beelen et al. 2008), and sex and educational level (Pope et al. 2002). The assessment of exposure to air pollution is a challenge, with some previous studies using centrally monitored concentration assigned to all persons living within a wide area around the monitoring station (Dockery et al. 1993; Filleul et al. 2005; Pope et al. 1995; Vineis et al. 2006), whereas other methods were based on a higher spatial resolution (Beelen et al. 2008; Nafstad et al. 2003; Nyberg et al. 2000). Further, some studies estimate exposure relating to a short period in time, for example, time of enrollment into a cohort (Dockery et al. 1993; Pope et al. 1995; Vineis et al. 2006), whereas other studies use residential addresses over longer time periods as the basis for assessing exposure (Nafstad et al. 2003; Nyberg et al. 2000).

In the study reported here, we tested the hypothesis that exposure to air pollution from traffic increases the risk for lung cancer. We used data from a large Danish cohort and applied detailed data on traffic and a dispersion model with high spatial resolution to calculate the concentrations of air pollution at the actual residential addresses over a 30-year period.

## Materials and Methods

### Design and study participants

The Danish Diet, Cancer and Health cohort study formed the base of the present study. During 1993–1997, 57,053 men and women 50–64 years of age living in Copenhagen and Aarhus areas were recruited into the Diet, Cancer and Health cohort study (Tjonneland et al. 2007). The baseline examination included a self-administered questionnaire on diet, smoking habits (status, intensity, and duration), occupational history, length of school attendance, and a number of other health-related items. We calculated smoking intensity by equating a cigarette to 1 g, a cheroot or a pipe to 3 g, and a cigar to 5 g of tobacco.

We followed up each cohort member for cancer occurrence until 27 June 2006 in the Danish Cancer Registry (Storm et al. 1997) and the Danish Pathology Data Bank (Herlev, Denmark) by use of the personal identification number, which is unique for each Danish citizen. We traced the date of death, emigration, or disappearance of cohort members in the Central Population Registry (Copenhagen, Denmark) by use of the personal identification number and retrieved the addresses of each participant from 1 January 1971 until 27 June 2006 from the same registry. We noted the dates of moving into and leaving each address and linked the addresses to the Danish address database to obtain geographic coordinates (denoted as “geocodes”), which were obtained for 94% of the addresses. Relevant Danish ethical committees and data protection agencies approved the study, and written informed consent was obtained from all participants.

The 241 cases diagnosed with lung cancer before 16 February 2001 among the Diet, Cancer and Health cohort members were included in a previous case–cohort study on air pollution and risk for lung cancer where we pooled data from three Danish cohorts (Raaschou-Nielsen et al. 2010). The present classical cohort study extends the follow-up period until 27 June 2006 and includes an additional 351 lung cancer cases.

### Exposure assessment

We calculated the outdoor concentration of NO_x_ and NO_2_ for each year at each residential address at which the cohort members had lived by use of the Danish AirGIS modeling system (http://www.dmu.dk/en/air/models/airgis/; see also Jensen et al. 2001), which is based on a geographic information system (GIS) and is used for estimating traffic-related air pollution with high temporal and spatial resolution. AirGIS can be used for a large number of addresses and to calculate air pollution at a location as the sum of three contributors: *a*) local air pollution from street traffic, calculated with the operational street pollution model (OSPM) from input data on traffic (intensity and type), emission factors for the car fleet, street and building geometry, and meteorology when modeling dispersion of tail pipe emissions in the street (http://www.dmu.dk/en/air/models/ospm; see also Berkowicz 2000); *b*) urban background, calculated from a simplified area source dispersion formula that takes into account urban vehicle emission density, city dimensions (transport distance), and building height (initial dispersion height) (Berkowicz et al. 2008); and *c*) regional background, estimated from trends at rural monitoring stations and from national vehicle emissions (Jensen 1998).

We established input data for the AirGIS system from various sources and integrated these data into the model. We developed a GIS road network, including construction year and traffic data for the period 1960–2005 (Jensen et al. 2009a), and built a database of emission factors for the Danish car fleet, with data on light- and heavy-duty vehicles back to 1960, which was entered into the emission module of the OSPM. We collected traffic data for the mid-1990s to coincide with the period of enrollment into the cohort and extrapolated forward and backward in time based on knowledge of historical traffic trends for different types of streets. Thus, we would expect less precision in the traffic data in the early than in the later time period. Similarly, we expect less precision for emission factors for the Danish car fleet in the early time period. We supplemented the national topographic GIS database of buildings with the construction year and building height from the national Building and Dwelling Register (Copenhagen, Denmark), which provided the correct street and building geometry for a given year at a given address. The geocode of an address refers to the location of the front door with a precision within 5 m for most addresses. With the geocode of an address and a specified year as the starting point, the AirGIS system automatically generates street configuration data for the OSPM, including street orientation, street width, building heights in wind sectors, and traffic amount, speed, and type, as well as other data required as input for the modeling system. Air pollution is calculated at 2 m height at the façade of the address building. The AirGIS system has been validated in several studies (Berkowicz et al. 2008; Jensen et al. 2001, 2009b; Raaschou-Nielsen et al. 2000), and the correlation between modeled and measured half-year mean NO_2_ concentrations at 204 positions in the greater Copenhagen area showed a correlation coefficient (*r*) of 0.90, with measured concentrations being on average 11% lower than modeled concentrations (Berkowicz et al. 2008). We also compared modeled and measured 1-month mean concentrations of NO_x_ and NO_2_ over a 12-year period (1995–2006) in a busy street in Copenhagen (Jagtvej, 25,000 vehicles/day, street canyon), which showed correlation coefficients (*r*) of 0.88 for NO_x_ and 0.67 for NO_2_. The modeled mean concentration over the whole 12-year period was 6% lower than the measured concentrations for NO_x_ and 12% lower for NO_2_ (Ketzel M, unpublished observations). Thus, the model well predicted both geographic and temporal variation. The AirGIS system calculates air pollution hour by hour, which in the present study we summarized as the yearly average concentration at each residential address. We focused on the concentration of NO_x_ as an indicator for air pollution from traffic because NO_x_ correlates strongly with other traffic-related pollutants, especially ultrafine PM: *r* = 0.93 for total particle number concentration (size, 10–700 nm) and *r* = 0.70 for PM_10_ (Hertel et al. 2001; Ketzel et al. 2003). We calculated the time-weighted average NO_x_ and NO_2_ concentrations at all addresses from 1 January 1971 and entered these concentrations as time-dependent variables into the statistical cancer risk model. The participants moved on average 2.4 times between 1 January 1971 and enrollment and on average 0.3 times between enrollment and censoring. If air pollution could not be calculated because of failed geocoding of an address, we imputed the concentration calculated at the preceding address. If the air pollution concentration was missing for the first address, we imputed the value at the subsequent address. We included only participants for whom the residential addresses were known and geocoded for ≥ 80% of the time from 1 January 1971 until censoring, that is, persons for whom air pollution concentrations were imputed for < 20% of time. We also estimated exposure in different time windows. However, the NO_x_ concentration calculated for the whole exposure period correlated strongly with that calculated after inclusion of a 10-year lag (*r* = 0.98) and that calculated as an annual average for the address at the time of enrollment (*r* = 0.86), respectively.

We used the geocodes of the address at the time of enrollment into the cohort and the GIS road network with traffic data to form two variables indicating the amount of traffic near the residence. The first variable was a dichotomous indicator for presence of a street with a traffic density > 10,000 vehicle/day within 50 m of the residence. The second variable summarized the total amount of kilometers driven by vehicles within 200 m of the residence each day as the product of street length and traffic density added up for all street lines within a 200-m circle around the address.

The three exposure indicators correlated moderately with correlation coefficients of 0.53 between calculated NO_x_ and presence of a major road within 50 m, 0.43 between calculated NO_x_ and traffic load within 200 m, and 0.43 between presence of a major road within 50 m and traffic load within 200 m.

The Danish AirGIS modeling system cannot provide reliable estimates for historical PM concentrations because the required input data on historical urban background concentrations and historical emission factors for the Danish car fleet are not available.

### Statistical methods

The end point for the risk analyses was primary lung cancer. Incidence rate ratios (IRRs) for lung cancer were estimated by a Cox proportional hazards model. We calculated two-sided 95% confidence intervals (CIs) on the basis of the Wald test statistic for regression parameters in Cox regression models. Age was the time scale, which ensured that the risk estimates were based on comparisons of individuals at exactly the same age, and analyses were corrected for delayed entry at the time of enrollment. People diagnosed with a malignant neoplasm, precancerous lesions of the cervix uteri (dysplasia, carcinoma *in situ*), nonmelanoma skin cancer, or benign central nervous system tumor before entry were excluded from the analyses. Censoring occurred at the time of death, loss of follow-up due to emigration or disappearance, the time of a cancer diagnosis, or 27 June 2006 (end of follow-up), whichever came first. We considered *p*-values < 5% to indicate statistical significance.

Data were analyzed both crude and adjusted for covariates defined at the time of enrollment: smoking status [never, former (with no requirement of a minimum nonsmoking period), present], smoking intensity (lifetime average, linear), duration of smoking (linear), length of school attendance (< 8, 8–10, and > 10 years), ETS (dichotomous, no/low, i.e., “no smoker in the home and ETS at work for less than 4 hr/day” vs. high), dietary intake of fruit (linear) as defined previously (Hansen et al. 2010), and a dichotomous variable indicating if ever worked for at least 1 year in an industry or job associated with risk for lung cancer: mining, electroplating, manufacturing of shoe/leather products, metal processing (welding/painting), foundry/steel rolling mill, shipyard, glass industry, building industry (roof constructor/asphalt worker/demolition worker), truck/bus/taxi driver, manufacturing of asbestos/cement, asbestos insulation, cement article industry, china and pottery industry, butcher, painter, welder, auto mechanic, waiter, or cook.

We formed four intervals for exposure to NO_x_, exposure to NO_2_, and traffic load within 200 m of the residence using the 25th, 50th, and 75th percentiles for all participants as the cutoff points and estimated the IRR for lung cancer for the higher exposure ranges compared with the lowest exposure range. We also estimated the IRRs as linear trends per 100-μg/m^3^ increment in NO_x_ concentration, per 10-μg/m^3^ change in NO_2_ concentration, and per 10^4^ vehicle km/day traffic load within 200 m of the residence. We also estimated the linear trend in association with the annual average NO_x_ concentration (modeled as a continuous variable) at the address at the time of enrollment. We analyzed the effect of exposure in separate models within strata of sex, smoking status, length of school attendance, and fruit consumption and compared the linear NO_x_ estimates between the strata using the Wald test.

## Results

Among the 57,053 cohort members, we excluded 571 with a malignant neoplasm, precancerous lesions of the cervix uteri (dysplasia, carcinoma *in situ*), nonmelanoma skin cancer, or a benign central nervous system tumor before enrollment; 2 because of uncertain date of cancer diagnosis; 960 for which their address history was not available in the Central Population Registry or their baseline address could not be geocoded; 1,365 because of missing data in potential confounders; and 1,185 because exposure was assessed for < 80% of the time from 1 January 1971 until diagnosis or censoring. We followed the included 52,970 cohort members up for on average 9.6 years and identified 592 lung cancer cases, corresponding to an incidence rate of 116 per 100,000 person-years.

Table 1 lists the characteristics of the cohort members and the cases. About half of the participants were men among both cases and cohort members. All smoking variables showed higher exposure among cases than among cohort members, including proportion of smokers at enrollment, smoking intensity, duration of smoking, and exposure to ETS. The proportion of participants with < 8 years of school attendance and with a low intake of fruit (< 100 g/day) was higher among cases than among the cohort members as a whole, and a higher proportion of cases had worked in an industry or job associated with a higher risk for lung cancer. All three air pollution variables showed higher exposure levels among cases than among the cohort members. Table 1 also shows that those living at addresses with high NO_x_ levels tended to have shorter school attendance and were more likely to be a smoker, to be exposed to ETS, to have a lower fruit intake, and to have worked in an industry or job associated with higher risk for lung cancer.

Table 2 shows that the crude lung cancer incidence rate was nine times higher among smokers than among nonsmokers, about twice as high among individuals with < 8 years compared with ≥ 8 years of school attendance and among those with low compared with a high fruit intake, and similar for men and women.

The highest quartile of time-weighted residential NO_x_ concentration was associated with an adjusted IRR for lung cancer of 1.30 (95% CI, 1.05–1.61), but associations did not follow a linear exposure–response pattern (Table 3). The adjusted IRR for lung cancer in association with living within 50 m of a major road (> 10,000 vehicles/day) at the time of enrollment was 1.21 (95% CI, 0.95–1.55). The highest quartile of total traffic load within 200 m of the residence at enrollment showed a nonsignificant association with lung cancer after adjustment. In general, associations were stronger in the crude analyses. The attenuation of the relative risk estimates in the adjusted analysis was almost entirely due to adjustment for active smoking.

After adjustment for potential confounders, the second, third, and fourth quartiles of NO_2_ exposure were associated with IRRs for lung cancer of 1.08 (95% CI, 0.84–1.39), 1.01 (95% CI, 0.80–1.28), and 1.12 (95% CI, 0.90–1.38), corresponding to a linear trend of 1.08 (95% CI, 0.94–1.24) per 10 μg/m^3^.

When considering the continuous NO_x_ and traffic load variables and the highest versus the lowest exposure categories for all three exposure markers, the IRRs for lung cancer were stronger among nonsmokers compared with smokers, although the interactions for the continuous variables were not statistically significant (Table 4). The IRRs were statistically significant among nonsmokers for the highest NO_x_ exposure category (IRR = 1.91; 95% CI, 1.10–3.30) and for presence of a major road within 50 m of the residence (IRR = 1.83; 95% CI, 1.04–3.23). The results showed a consistent pattern of higher IRRs for lung cancer in association with the continuous NO_x_ and traffic load variables and the highest versus the lowest exposure categories of all three markers of traffic-related air pollution among cohort members with ≥ 8 years of school attendance compared with ≤ 7 years. The *p*-values for interaction were 0.03 and 0.04 for the linear trends for NO_x_ and traffic load, respectively. Table 4 also shows higher relative risk for lung cancer in association with the continuous NO_x_ variable, the highest versus the lowest NO_x_ exposure categories, and presence of a major road within 50 m among cohort members with a dietary intake of < 100 g/day fruit than among those eating > 100 g/day fruit, but the interaction for the continuous NO_x_ variable was not significant (*p* = 0.67).

## Discussion

This study showed associations between different markers of air pollution from traffic at the residence and risk for lung cancer in a prospective cohort study. The results indicated stronger associations among nonsmokers, among participant with longer school attendance, and among those with relatively low dietary intake of fruit.

We used a large prospective cohort where information on potential confounding factors was collected at enrollment, that is, with no potential for recall bias. We ensured complete follow-up for cancer and vital status by using the population-based Danish Cancer Registry, the Danish Pathology Data Bank, and the Danish Central Population Registry. The Central Population Registry also provided information on residential addresses back to 1971, which we used in the exposure assessment.

The exposure assessment is a major challenge in studies of health effects of long-term exposure to air pollution. In the present study we used three markers for air pollution from traffic at the residences, which were moderately correlated (*r* between 0.43 and 0.53). We calculated the outdoor NO_x_ level for all addresses over decades using a validated model requiring comprehensive input data, and the two other markers are simple, intuitively understandable measures of traffic at the residence at the time of enrollment, with one referring to presence of a major road within 50 m of the residence and the other summarizing the total traffic load within 200 m of the residence. Although the calculated NO_x_ concentration is the marker that takes into account most factors influencing the long-term NO_x_ concentration, to our knowledge no studies have determined whether such comprehensive modeling of the concentration at the front door in fact reflects personal exposure of the individuals living at the address better than simpler indicators for traffic in the neighborhood. To properly illuminate which of the three exposure markers best reflects personal exposure of persons living at the address would require measurements of personal exposure as the gold standard for comparison with each of the exposure markers.

Exposure over a long period, perhaps over a whole life, is probably relevant for the development of lung cancer, and the present study benefited from information on residential histories from 1971 onward as the basis for the assessment of exposure to NO_x_. One of the few previous studies with information on exposure decades back in time indicated that the effect of air pollution on the risk for lung cancer is stronger after inclusion of a lag, that is, after disregarding exposure during the period closest to the diagnosis (Nyberg et al. 2000). In the present study, however, the NO_x_ concentration calculated for the whole exposure period correlated strongly with both the NO_x_ concentration calculated after inclusion of a 10-year lag (*r* = 0.98) and that calculated as an annual average for the address at the time of enrollment (*r* = 0.86). Thus, our material would seem not to be suitable for investigating possible effects of timing of exposure. The high correlation between NO_x_ averages for all addresses since 1971 and the annual mean of NO_x_ at the residence at time of enrollment indicates that the exposure markers based on the address at the time of enrollment in the present study might reflect exposure for a much longer time period.

The dispersion models we used to assess NO_x_ and NO_2_ levels at the addresses of study participants have been successfully validated (Berkowicz et al. 2008; Raaschou-Nielsen et al. 2000) and applied both in Denmark (Raaschou-Nielsen et al. 2001) and in the United States (Jensen et al. 2009b). Also, we used indicators for traffic near the residence. Such markers of air pollution concentrations are inevitably associated with some degree of uncertainty. We cannot see how this uncertainty could depend on development of lung cancer, however, and such nondifferential misclassification would only in rare situations create artificial associations (Dosemeci et al. 1990).

Our finding of associations between indicators of air pollution from traffic and risk for lung cancer is in accordance with findings of previous studies showing effects of NO_2_ or proximity to traffic (Beelen et al. 2008; Filleul et al. 2005; Nafstad et al. 2003; Nyberg et al. 2000; Vineis et al. 2006). A previous cohort study, conducted in Norway, that used NO_x_ as an indicator of air pollution (Nafstad et al. 2003) showed a risk ratio for lung cancer of 1.36 (95% CI, 1.01–1.83) in association with ≥ 30 μg/m^3^ NO_x_ at the residence compared with < 10 μg/m^3^ NO_x_. That is similar to the rate ratio of 1.30 (95% CI, 1.05–1.61) in association with ≥ 30 μg/m^3^ NO_x_ at the residence compared with < 17 μg/m^3^ NO_x_ that we found in the present study. Further, a previous case–cohort study combining data from three Danish cohorts and including 241 of the 592 cases in the present study showed an IRR of 1.37 (95% CI, 1.06–1.76) per 100 μg/m^3^ NO_x_ (linear trend) (Raaschou-Nielsen et al. 2010), which is higher than the IRR of 1.09 (95% CI, 0.79–1.51) we found in the present study, although the CIs widely overlapped.

Two previous studies have investigated the risk for lung cancer in association with residence near heavy-traffic roads. Identical risk estimates for proximity to traffic cannot be expected in different studies because of different definitions of this exposure variable and differences in study populations. Nevertheless, Vineis et al. (2006) found an odds ratio of 1.46 (95% CI, 0.89–2.40) and Beelen et al. (2008) found an IRR of 1.11 (95% CI, 0.91–1.34), which are both comparable with the IRR of 1.21 (95% CI, 0.95–1.55) that we found in the present study.

Although associations have been found with NO_x_ and NO_2_ in the present and previous studies, these single pollutants should be considered indicators for vehicle engine exhaust, which is a complex mixture including many carcinogenic and mutagenic chemicals (IARC 1989). NO_x_ has been shown to correlate closely with PM, especially the ultrafine fraction emitted from diesel engines in Danish streets (Hertel et al. 2001; Ketzel et al. 2003). It is difficult to disentangle the effect of single air pollutants in epidemiologic designs because they are part of complex mixtures, but it seems likely that for cancer risk PM from traffic emissions is most important. This comprises mainly PM with ultrafine size, large surface area, and absorbed polycyclic aromatic hydrocarbons, transition metals, and other substances causing oxidative stress, inflammation, and direct and indirect genotoxicity (Borm et al. 2004; Moller et al. 2010). However, although NO_2_ is not known as genotoxic, it cannot be excluded that NO_2_ can act as a tumor promoter in relation to diesel exhaust particles, as shown in an animal carcinogenicity study (Ohyama et al. 1999). In the present study, we focused on air pollution from traffic, which is the major source of NO_x_ air pollution in Danish cities. Two other Scandinavian studies showed effects of traffic-related NO_x_ but not of heating and industry-related SO_2_ on the risk for lung cancer (Nafstad et al. 2003; Nyberg et al. 2000).

The relative risk estimates in the present study were substantially attenuated by adjustment for active smoking, consistent with smoking being a strong risk factor for lung cancer and smoking being associated with residence at locations with high air pollution levels (Table 1). However, we found stronger relative risks for lung cancer in association with air pollution among nonsmokers than among smokers, indicating that residual confounding by smoking is not the explanation for the observed associations between traffic pollution and risk for lung cancer. The higher relative risk among nonsmokers is in accordance with the results of previous studies (Beelen et al. 2008; Nyberg et al. 2000; Pope et al. 2002; Yorifuji et al. 2010). The lung cancer incidence rate is much lower among nonsmokers than among smokers (Table 2), and a higher relative risk among nonsmokers therefore does not necessarily correspond to a higher absolute risk. Thus, the 91% higher relative risk for the upper air pollution exposure group among nonsmokers would correspond to 27 excess lung cancer cases per 100,000 person-years (0.91 × 30 per 100,000 person-years), whereas the 21% higher relative risk among smokers would correspond to 57 extra cases per 100,000 person-years (0.21 × 270 per 100,000 person-years). Nevertheless, the wide CIs around the risk estimates indicate that the different relative risk estimates for nonsmokers and present smokers should be interpreted with caution.

The results of this and a previous study (Beelen et al. 2008) indicate that an association between air pollution and risk for lung cancer might be present mainly among individuals with a low fruit consumption. An expert panel established by the World Cancer Research Fund and the American Institute for Cancer Research concluded in 2007 that fruits probably protect against lung cancer (World Cancer Research Fund/American Institute for Cancer Research 2007). Among the possible mechanisms are the scavengers of free radicals and reactive oxygen species, as well as possible up-regulation of protective enzymes by constituents present in fruits, protecting against oxidative damage to the DNA (Moller and Loft 2006). The suggested modification by fruit intake of an effect of air pollution on the risk for lung cancer might similarly be explained by the presence of oxidants and prooxidants in air pollution (Borm et al. 2004; Moller et al. 2010), which might lead to a higher risk for lung cancer mainly in individuals with low intake of antioxidants in fruits. A high intake of fruits might also protect against lung cancer by reducing the formation of DNA adducts from polycyclic aromatic hydrocarbons (Palli et al. 2004; Peluso et al. 2000). Fruit consumption might also be a marker of other characteristics, which might have contributed to the results.

The present study showed a stronger association between markers of air pollution and risk for lung cancer among individuals with ≥ 8 years of school attendance. The opposite tendency was observed in a previous study from the United States (Pope et al. 2002), although the pattern was less clear in a recent analysis with a longer follow-up period (Krewski et al. 2009). A Dutch study showed no clear modification of the association by educational level (Beelen et al. 2008). It is obvious that educational level per se does not influence the risk for lung cancer. Instead, a variety of lifestyle and behavioral factors, other exposures, and overall health that are associated with educational level might influence lung cancer risk. Associations between educational level and risk (or protective) factors for lung cancer might well differ by country and time period, possibly explaining the heterogeneity of these results. The apparent effect modification in the present study might also be due to chance.

There is a partial overlap in the case series of this and a previous lung cancer study (Raaschou-Nielsen et al. 2010). The present study, however, differs from the previous in many aspects, among which are the longer follow-up (and hence most new cases), the classical cohort design with exposure assessment for all 52,970 included cohort members, inclusion of two traffic exposure variables, and inclusion of ETS, occupation, and fruit intake as potential confounders or effect modifiers. Moreover, the classical cohort design of the present study facilitated calculation of absolute lung cancer rates in strata defined by potential effect modifiers, which qualifies the discussion about different effects of air pollution between smokers and nonsmokers. The overall conclusions of this and the previous study are very similar.

In conclusion, this study showed associations between risk for lung cancer and different markers of air pollution from traffic near the residence, in line with the weight of the epidemiologic evidence to date.

## Figures and Tables

**Table 1:** Characteristics of all study participants, cases, and those with low and high levels of NO_x_ at their residences.

	Cohort		Cases		NO_x_ < 29.7 μg/m^3^		NO_x_ ≥ 29.7 μg/m^3^	
Characteristic	*n* (%)	Mean/median (5th–95th percentile)	*n* (%)	Mean/median (5th–95th percentile)	*n* (%)	Mean/median (5th–95th percentile)	*n* (%)	Mean/median (5th–95th percentile)
All participants	52,970 (100)		592 (100)		39,750 (75.0)		13,220 (25.0)	

Age at enrollment (years)		56.6/56.1 (50.7–64.1)		58.5/59.1 (51.1–64.7)		56.6/56.1 (50.7–64.1)		56.7/56.2 (50.7–64.1)

Sex								
Male	25,182 (47.5)		300 (50.7)		19,003 (47.8)		6,179 (46.7)	
Female	27,788 (52.5)		292 (49.3)		20,747 (52.2)		7,041 (53.3)	

School attendance (years)								
< 8	17,490 (33.0)		298 (50.3)		12,806 (32.2)		4,684 (35.4)	
≥ 8	35,480 (67.0)		294 (49.7)		26,944 (67.8)		8,536 (64.6)	

Smoking								
Nonsmoker	33,717 (63.6)		100 (16.9)		26,469 (66.6)		7,248 (54.8)	
Present smoker	19,253 (36.4)		492 (83.1)		13,281 (33.4)		5,972 (45.2)	
Intensity (g/day)^a^		16.3/14.8 (3.8–34.4)		19.5/18.2 (9.3–35.5)		16.1/14.6 (3.6–34.4)		16.7/15.3 (4.2–34.3)
Duration (years)^a^		29.8/33.0 (7.0–46.0)		39.5/40.0 (29.0–49.0)		29.3/32.0 (7.0–46.0)		31.3/34.0 (9.0–46.0)

ETS								
No/low	18,939 (35.8)		52 (8.8)		15,109 (38.0)		3,830 (29.0)	
High	34,031 (64.2)		540 (91.2)		24,641 (62.0)		9,390 (71.0)	

Fruit intake (g/day)								
< 100	17,929 (33.8)		283 (47.8)		13,060 (32.9)		4,869 (36.8)	
≥ 100	35,041 (66.2)		309 (52.2)		26,690 (67.1)		8,351 (63.2)	

Risk occupation^b^								
No	38,143 (72.0)		354 (59.8)		28,966 (72.9)		9,177 (69.4)	
Yes	14,827 (28.0)		238 (40.2)		10,784 (27.1)		4,043 (30.6)	

NO_x_ at front door^c^ (μg/m^3^)		28.3/21.8 (14.8–68.9)		31.7/24.0 (15.2–77.7)				

Major road^d^ within 50 m								
No	48,628 (91.8)		520 (87.8)					
Yes	4,342 (8.2)		72 (12.2)					

Traffic load within 200 m (10^3^ vehicle km/day)		4.7/2.6 (0.3–15.3)		5.5/3.7 (0.3–15.7)				

a Based on all ever-smokers.

b Ever worked for at least 1 year in an industry or job associated with higher risk for lung cancer (see “Materials and Methods” for specification).

c Time-weighted average for the period 1 January 1971 to the censoring date.

d More than 10,000 vehicles/day.

**Table 2:** Crude lung cancer incidence rates by sex, smoking, length of school attendance, and fruit intake (based on data collected at enrollment of 52,970 cohort members).

Characteristic	Person-years at risk	No. of cases	Crude lung cancer incidence rate per 100,000 person-years
Sex			
Male	241,153	300	124
Female	269,751	292	108

Smoking			
Nonsmoker	327,836	100	30
Present smoker	183,068	492	270

School attendance (years)			
< 8	168,332	298	177
≥ 8	342,572	294	86

Fruit intake (g/day)			
< 100	172,256	283	164
≥ 100	338,648	309	91

**Table 3:** IRRs for lung cancer associated with the concentration of NO_x_ and proximity to traffic at the residence (based on 52,970 cohort members, 592 lung cancer cases, and 510,904 person-years at risk).

	IRR (95% CI)	
Air pollution indicator	Crude	Adjusted^a^
NO_x_ concentration (μg/m^3^)^b^,^c^		
< 17.2	1.00	1.00
17.2 – 21.8	1.25 (0.97–1.62)	1.09 (0.84–1.40)
21.8 – 29.7	0.92 (0.73–1.17)	0.93 (0.73–1.18)
> 29.7	1.58 (1.27–1.97)	1.30 (1.05–1.61)
Linear trend per 100 μg/m^3^	1.53 (1.13–2.07)	1.09 (0.79–1.51)
Linear trend per 100 μg/m^3^ at enrollment^d^	1.47 (1.09–2.00)	1.06 (0.77–1.46)

Major road^e^ within 50 m		
No	1.00	1.00
Yes	1.47 (1.15–1.88)	1.21 (0.95–1.55)

Traffic load within 200 m (10^3^ vehicle km/day)^c^		
< 0.88	1.00	1.00
0.88–2.61	1.09 (0.85–1.40)	0.98 (0.76–1.27)
2.61–6.73	1.30 (1.02–1.66)	1.05 (0.83–1.34)
> 6.73	1.60 (1.27–2.02)	1.17 (0.92–1.47)
Linear trend per 10^4^ vehicle km/day	1.21 (1.06–1.38)	1.03 (0.90–1.19)

a Adjusted for smoking (status, intensity, duration), ETS, length of school attendance, fruit intake, and employment in an industry or job associated with higher risk for lung cancer (see “Materials and Methods” for specification). We adjusted for age by using it as time scale in the Cox model. Because of exclusion of cohort members with a missing value in any covariate, the number of persons is identical in the crude and the adjusted analyses.

b Time-weighted average concentration of NO_x_ at residences from 1 January 1971 until censoring.

c The cutoff points between exposure groups were the 25th, 50th, and 75th percentiles for all participants.

d One-year average concentration of NO_x_ at the address at enrollment.

e More than 10,000 vehicles/day.

**Table 4:** Adjusted^a^ IRRs (95% CIs) for lung cancer in association with indicators for air pollution from traffic, by sex, smoking, length of school attendance, and fruit intake (based on 52,970 cohort members, 592 lung cancer cases, and 510,904 person-years at risk).

	Sex		Smoking status		Length of school attendance (years)		Fruit intake (g/day)	
Air pollution indicator	Male	Female	Nonsmoker	Present smoker	< 8	≥ 8	< 100	≥ 100
NO_x_ concentration (μg/m^3^)^b^,^c^								
< 17.2	1.00	1.00	1.00	1.00	1.00	1.00	1.00	1.00
17.2–21.8	1.14 (0.80–1.62)	0.95 (0.65–1.37)	1.07 (0.59–1.94)	1.09 (0.82–1.45)	0.88 (0.62–1.25)	1.38 (0.95–2.01)	1.13 (0.77–1.66)	1.05 (0.75–1.48)
21.8–29.7	0.97 (0.70–1.34)	0.85 (0.60–1.20)	0.83 (0.46–1.51)	0.95 (0.73–1.23)	1.12 (0.81–1.56)	0.76 (0.54–1.08)	0.84 (0.59–1.19)	1.01 (0.73–1.39)
> 29.7	1.16 (0.85–1.57)	1.45 (1.06–1.99)	1.91 (1.10–3.30)	1.21 (0.95–1.45)	0.99 (0.73–1.34)	1.71 (1.25–2.33)	1.55 (1.12–2.13)	1.10 (0.82–1.49)
Linear trend per 100 μg/m^3^	1.03 (0.63–1.67)	1.03 (0.66–1.60)	1.51 (0.72–3.16)	1.02 (0.71–1.46)	0.73 (0.43–1.23)	1.53 (1.01–2.30)	1.26 (0.82–1.56)	0.89 (0.54–1.45)
*p*-Value for interaction^d^	0.99		0.34		0.03		0.67	

Major road^e^ within 50 m								
No	1.00	1.00	1.00	1.00	1.00	1.00	1.00	1.00
Yes	1.27 (0.90–1.80)	1.09 (0.77–1.54)	1.83 (1.04–3.23)	1.12 (0.85–1.47)	1.04 (0.72–1.50)	1.40 (0.99–1.96)	1.35 (0.96–1.89)	1.07 (0.75–1.54)

Traffic load within 200 m (10^3^ vehicle km/day)^c^								
< 0.88	1.00	1.00	1.00	1.00	1.00	1.00	1.00	1.00
0.88–2.61	0.93 (0.66–1.31)	1.03 (0.70–1.50)	1.22 (0.70–2.13)	0.93 (0.70–1.23)	0.79 (0.56–1.14)	1.23 (0.85–1.75)	0.90 (0.62–1.30)	1.07 (0.76–1.51)
2.61–6.73	0.96 (0.69–1.34)	1.07 (0.75–1.53)	0.91 (0.50–1.65)	1.08 (0.82–1.40)	0.97 (0.70–1.35)	1.14 (0.80–1.63)	1.03 (0.73–1.47)	1.06 (0.76–1.48)
> 6.73	1.08 (0.79–1.49)	1.11 (0.78–1.57)	1.20 (0.69–2.09)	1.15 (0.89–1.49)	0.92 (0.67–1.27)	1.47 (1.05–2.06)	1.15 (0.82–1.61)	1.16 (0.84–1.60)
Linear trend per 10^4^ vehicle km/day	1.01 (0.83–1.24)	0.96 (0.79–1.17)	1.21 (0.88–1.67)	1.00 (0.86–1.16)	0.89 (0.72–1.09)	1.18 (0.98–1.42)	1.00 (0.81–1.22)	1.06 (0.88–1.28)
*p*-Value for interaction^d^	0.70		0.30		0.04		0.65	

a Adjusted for smoking (status, intensity, duration), ETS, length of school attendance, fruit intake, and employment in an industry or job associated with higher risk for lung cancer (see “Materials and Methods” for specification), but no adjustment for the stratification variable. We adjusted for age by using it as time scale in the Cox model.

b Time-weighted average concentration of NO_x_ at residences from 1 January 1971 until censoring.

c The cutoff points between exposure groups were the 25th, 50th, and 75th percentiles for all participants.

d Test of the null hypothesis that the linear trends are identical.

e More than 10,000 vehicles/day.
